# Role of the gut microbiota in the pharmacological effects of traditional Kampo medicine and natural products

**DOI:** 10.1007/s11418-025-01959-7

**Published:** 2025-10-10

**Authors:** Nobutomo Ikarashi, Risako Kon, Hiroyasu Sakai, Tomoo Hosoe

**Affiliations:** 1 Department of Biomolecular Pharmacology, Hoshi University, 2-4-41 Ebara, Shinagawa-ku, Tokyo, 142-8501 Japan; 2 Department of Toxicology, Hoshi University, Tokyo, Japan

**Keywords:** Gut microbiota, Kampo medicines, Bofutsushosan, Daiokanzoto, Daikenchuto, Hangeshashinto

## Abstract

The human gastrointestinal tract harbors over a thousand coexisting bacterial species that together constitute a highly complex intestinal microbiome. Dysbiosis of this microbial ecosystem has been implicated in the pathogenesis of disorders such as ulcerative colitis and obesity and has driven the development of microbiota-targeted prevention and treatment strategies. Traditional Kampo medicines are used with the aim of restoring the balance of the whole body, having been developed and established uniquely in Japan based on traditional Chinese medicine with a history of over two millennia. Recent evidence has revealed that the gut microbiota plays critical roles in the pharmacological effects of traditional Kampo medicines. For example, the anti-obesity activity of Bofutsushosan has been linked to an increase in *Akkermansia muciniphila*. The laxative effect of Daiokanzoto is related to rheinanthrone, which is produced from sennoside A through the gut microbiota, and drugs and foods that can alter the gut microbiota change the laxative activity of Daiokanzoto. In recent years, clinical trials have been conducted to examine the relationship between changes in the gut microbiota and the pharmacological effects of Kampo medicines, and this relationship has come to the forefront. This review discusses the findings of basic and clinical research on the role of the gut microbiota in the pharmacological action of traditional Kampo medicines, focusing on Bofutsushosan, Daiokanzoto, Daikenchuto, and Hangeshashinto, and discusses future issues and prospects for research into these interactions.

**Graphical abstract:**

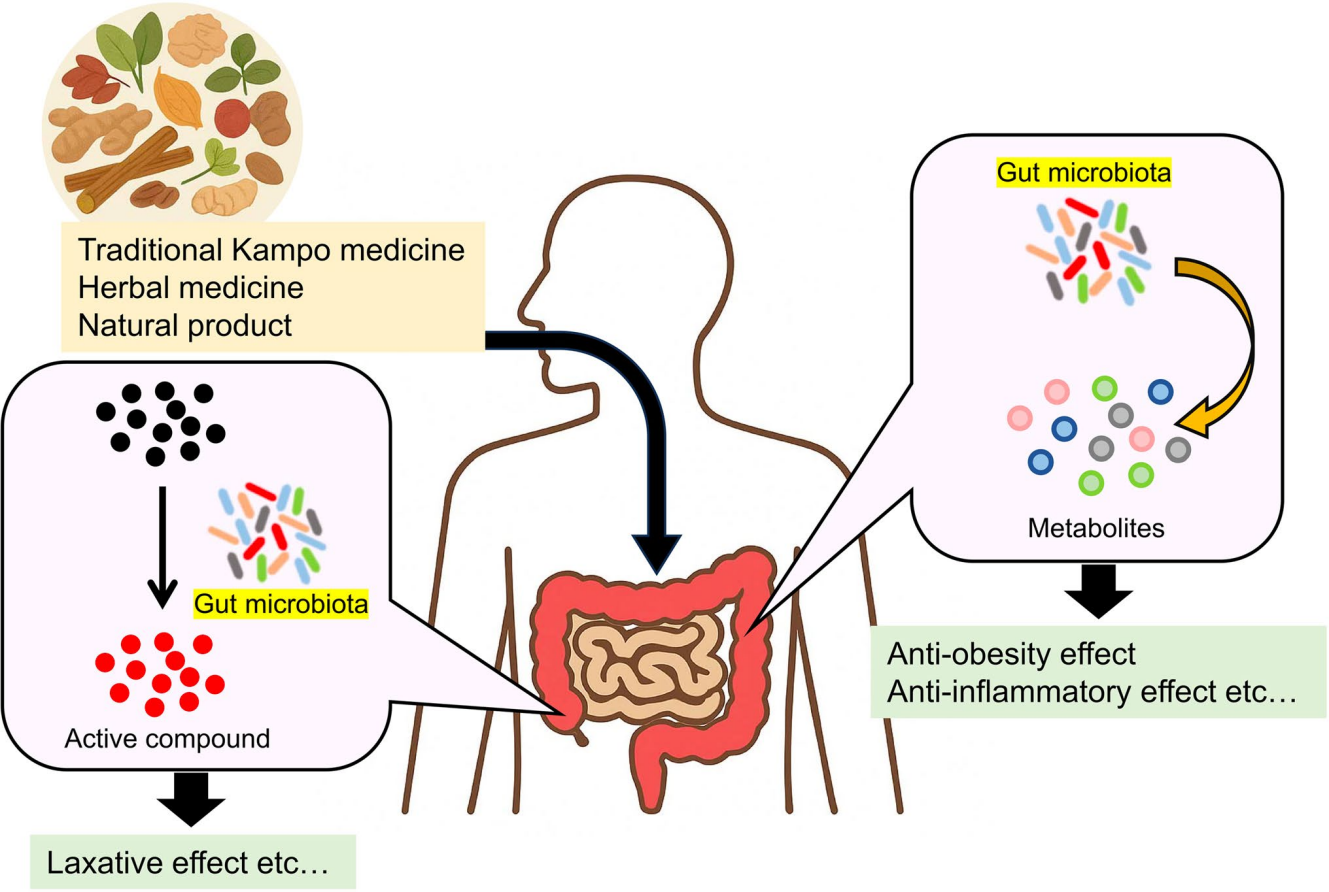

## Introduction

Traditional Kampo medicine is a combination of natural herbal medicines (materials derived from plants, animals, and minerals) designed to balance the entire body. Building upon thousands of years of clinical experience in traditional Chinese medicine, traditional Kampo medicine was developed and established in Japan as a therapeutic system aimed at restoring systemic balance. In other words, treatment with traditional Kampo medicines does not simply suppress symptoms temporarily but rather places emphasis on correcting overall imbalances, including the constitution, environment, and lifestyle of the patient. This approach is based on the theories of "yin-yang and the five elements" and "Qi, Blood, and Fluids", and it is positioned within the fundamental concept of Oriental medicine, which posits that stagnation or disharmony in the flow of vital energy (Qi) underlies disease pathogenesis.

As noted above, each Kampo formula comprises multiple crude-drug components rather than a single active ingredient; thus, its therapeutic effects are thought to arise from additive and synergistic interactions among these constituents. In recent years, advances in the extraction of active compounds, structural elucidation, and metabolic pathway analysis have begun to integrate centuries of empirical use with molecular‒biological insights, progressively establishing scientific evidence. Concurrently, some pharmacological actions of traditional Kampo formulations may be mediated via interactions with the gut microbiota. Moreover, not only traditional Kampo medicines but also individual crude drugs and other natural products are now recognized to engage in reciprocal relationships with the gut microbiota.

In this review, we describe the effects of traditional Kampo medicines, crude drugs, and natural products and their relationships with intestinal bacteria and discuss the prospects and challenges for future clinical application.

## Physiological functions of the gut microbiota and recent insights

The human intestine contains 1000 types and 100 trillion bacteria. Whereas earlier studies established that the gut microbiota influences host immune and metabolic functions, recent advances in 16S rRNA sequencing and metagenomic analysis have enabled the identification of diverse taxa that eluded conventional methods and have begun to elucidate the specific roles played by individual members of this complex ecosystem.

In addition, the relationship between intestinal bacteria and disease has also been shown, and control of the intestinal environment is becoming a new target for the prevention and treatment of various diseases. In fact, a clinical trial of fecal microbiota transplantation (FMT) aimed at improving intestinal bacterial imbalance (dysbiosis) for *Clostridioides difficile* infection (CDI) caused by long-term antibacterial use was conducted, and a high response rate was reported [1]. In addition, in 2023, the first oral microbial preparation (fecal microbiota spores, live-brpk) was approved by the U.S. Food and Drug Administration as a preventive drug for CDI. Clinical trials are also being conducted on microbial preparations for diseases other than CDI [2, 3].

These developments in elucidating the impact of gut microbes on human health have catalyzed efforts, particularly in Western countries, to translate microbiota research into novel preventive and therapeutic agents. Simultaneously, Japan and other Asian nations have initiated their own clinical trials of FMT, marking the transition of gut microbiology into a new, clinically oriented phase.

## Role of the gut microbiota in the pharmacological actions of Kampo formulations, crude drugs, and natural products

### Bofutsushosan

Bofutsushosan (BTS; Fang-Feng-Tong-Sheng-San in Chinese) is a Kampo formulation comprising 18 crude-drug components: *Angelicae acutilobae radix* (Japanese Angelica Root), *Paeoniae radix* (Peony Root), *Cnidii rhizoma* (Cnidium Rhizome), *Gardeniae fructus* (Gardenia Fruit), *Forsythiae fructus* (Forsythia Fruit), *Menthae herba* (Mentha Herb), *Zingiberis rhizoma* (Ginger), *Schizonepetae spica* (Schizonepeta Spike), *Saposhnikoviae radix* (Saposhnikovia Root and Rhizome), *Ephedrae herba* (Ephedra Herb), *Rhei rhizoma* (Rhubarb), *Sal mirabilis* (sodium sulfate hydrate), *Atractylodis rhizoma* (Atractylodes Rhizome), *Platycodi radix* (Platycodon Root), *Scutellariae radix* (Scutellaria Root), *Glycyrrhizae radix* (Glycyrrhiza), *Gypsum fibrosum* (Gypsum), and *Kasseki* (aluminum silicate hydrate with silicon dioxide). Its antiobesity effects have been attributed, in part, to the upregulation of uncoupling protein-1 in white adipose tissue [4] and the inhibition of intestinal lipid absorption [5].

Recent studies suggest that modulation of the gut microbiota also contributes to its efficacy. Nishiyama et al. reported that the administration of BTS to obese diabetic model mice significantly attenuated weight gain, improved serum lipid profiles, and reduced hepatic steatosis concomitant with a marked increase in *Akkermansia muciniphila* abundance [6]. Similarly, Fujisaka et al. demonstrated that BTS treatment in mice fed a high-fat diet elevated the levels of *A. muciniphila* [7]*. A. muciniphila*, a mucin-degrading bacterium that produces short-chain fatty acids such as acetate and propionate, is known to improve obesity [8]. Clinical supplementation with *A. muciniphila* has been shown to ameliorate obesity and insulin resistance in humans [9].

Taken together, these findings indicate that, in addition to its previously identified mechanisms, BTS may exert antiobesity effects in part by promoting the proliferation of *A. muciniphila*. Moreover, natural compounds such as capsaicin, found in *Capsici fructus* (Capsicum), have also been reported to suppress high-fat diet-induced weight gain while increasing *A. muciniphila* levels [10], highlighting the emerging importance of gut microbiota-mediated actions in the efficacy of both Kampo formulas and individual natural products.

### Daiokanzoto

Daiokanzoto (DKZT, Da-Huang-Gan-Cao-Tang in Chinese), a Kampo formulation composed solely of *Rhei rhizoma* (Rhubarb) and *Glycyrrhizae radix* (Glycyrrhiza), is traditionally prescribed for constipation. Its laxative effect is attributed principally to sennoside A, a dianthrone glycoside derived from Rhubarb.

Elke Leng-Peschlow first demonstrated in rats that the oral administration of sennoside A markedly enhances colonic peristalsis without affecting small intestinal motility [11]. Dreessen et al. subsequently reported that incubation of sennoside A with cecal contents generates rheinanthrone and that rheinanthrone itself stimulates colonic motility when it is administered to mice [12]. β-Glucosidase activity from *Bifidobacterium* spp. mediates the rate-limiting hydrolysis of sennoside A to rheinanthrone [13], and antibiotic pretreatment attenuates the laxative response to both sennoside A and DKZT, confirming the essential role of the gut microbiota [14]. Mechanistically, Kon et al. reported that rheinanthrone activates colonic macrophages, leading to downregulation of the water channel aquaporin-3 (AQP3) in the colonic epithelium [15, 16]. AQP3 normally facilitates osmotic water absorption from the lumen; its relationship with the laxative activity of laxatives [17, 18] and dietary fiber [19] has been clarified. Thus, the gut microbiota is essential for the laxative effect of DKZT.

Recent work has also elucidated how Glycyrrhiza modulates the activity of DKZT. Takayama et al. reported that the Glycyrrhiza constituents liquiritin and liquiritin apioside enhance microbial enzyme activity, thereby regulating the conversion of sennoside A to rheinanthrone [14, 20]. Although the laxative activity of Rhubarb decreases with continued use and resistance develops, Glycyrrhiza suppresses resistance to Rhubarb by regulating the changes in the gut microbiota caused by Rhubarb [21]. In addition, dietary changes that alter the gut microbiota can modulate the laxative efficacy of DKZT [22].

Collectively, these findings establish that the purgative action of DKZT is critically dependent on biotransformation of the gut microbiota and is subject to modulation by both coformulated herbal constituents and dietary or drugs.

### Daikenchuto

Daikenchuto (DKT, Da-Jian-Zhong-Tang in Chinese) is formulated from *Zingiberis rhizoma processum* (processed Ginger), *Zanthoxyli peperiti pericarpium* (Japanese Zanthoxylum peel), *Ginseng radix* (Ginseng), and *Saccharum granorum* (Koi, maltose) and is indicated for enhancing intestinal motility and treating postoperative paralytic ileus. Its prokinetic effects are mediated by the activation of transient receptor potential vanilloid 1 (TRPV1)/transient receptor potential ankyrin 1 (TRPA1) via hydroxy-α-sanshool from Japanese Zanthoxylum peel and [6]-shogaol from processed Ginger [23], as well as by the augmentation of intestinal blood flow through adrenomedullin secretion from intestinal epithelial cells [24–26].

Oral administration of DKT to mice has been shown to modulate the gut microbiota, resulting in increased luminal short-chain fatty acid levels [27]. Fecal samples from DKT-treated mice also demonstrated enhanced microbial conversion of ginsenoside Rb1 into the bioactive metabolite compound K [27]. In a dextran sulfate sodium (DSS)-induced colitis model, DKT ameliorated disease severity concomitant with increases in *Lactobacillus* spp. and elevated propionate production; propionate, in turn, stimulated colonic group 3 innate lymphoid cells to promote epithelial repair and host defense [28]. Furthermore, DKT enriched butyrate-producing bacteria, such as *Parabacteroides*, *Allobaculum*, and *Akkermansia*, which correlated with the upregulation of secretory leukocyte protease inhibitors and tight junction proteins, thereby mitigating colonic inflammation [29].

Hanada et al. conducted a prospective, randomized controlled trial of DKT in patients undergoing laparoscopic colectomy to assess postoperative abdominal symptoms. Although DKT did not significantly improve clinical abdominal outcomes, it reduced the relative abundance of the genera *Serratia* and *Bilophila* in the gut microbiota, demonstrating that DKT-induced microbiota shifts also occur in humans [30].

### Hangeshashinto

Hangeshashinto (HST, Ban-Xia-Xie-Xin-Tang) is a Kampo formula composed of *Zingiberis rhizoma processum* (processed Ginger), *Glycyrrhizae radix* (Glycyrrhiza), *Scutellariae radix* (Scutellaria root), *Coptidis rhizoma* (Coptis rhizome), *Ziziphi fructus* (Jujube), *Pinelliae tuber* (Pinellia tuber), and *Ginseng radix* (Ginseng) and is indicated for oral ulcers, diarrhea, and gastritis.

Yoshida et al. evaluated HST in patients with irritable bowel syndrome with diarrhea (IBS-D). Treatment significantly improved stool frequency, consistency, and abdominal pain and induced a marked shift in gut microbial β-diversity: *Bacteroides* and *Ruminococcus* decreased, whereas *Megasphaera* and *Subdoligranulum* increased [31]. These data demonstrate that HST exerts antidiarrheal effects at least in part via modulation of the gut microbiota.

In clinical practice, HST has proven effective against delayed diarrhea induced by the chemotherapeutic agent irinotecan (CPT-11) [32, 33]. SN-38 is a metabolite of CPT-11, which undergoes hepatic glucuronidation to SN-38G for biliary excretion; bacterial β-glucuronidase in the gut then deconjugates SN-38G back to SN-38, which damages the intestinal mucosa and precipitates diarrhea [34]. The absence of diarrhea in germ-free mice underscores the pivotal role of microbial β-glucuronidase in this process [35].

HST contains baicalein, an herbal medicine contained in Scutellaria root, which inhibits bacterial β-glucuronidase activity [36]. Although β-glucuronidase is also present in human intestinal epithelial cells, an inhibitor targeting β-glucuronidase in intestinal bacteria suppresses CPT-11-induced diarrhea [37]. Therefore, HST, through the action of baicalein, suppresses the deconjugation of SN-38G and thus inhibits diarrhea caused by CPT-11.

**Fig. 1:**
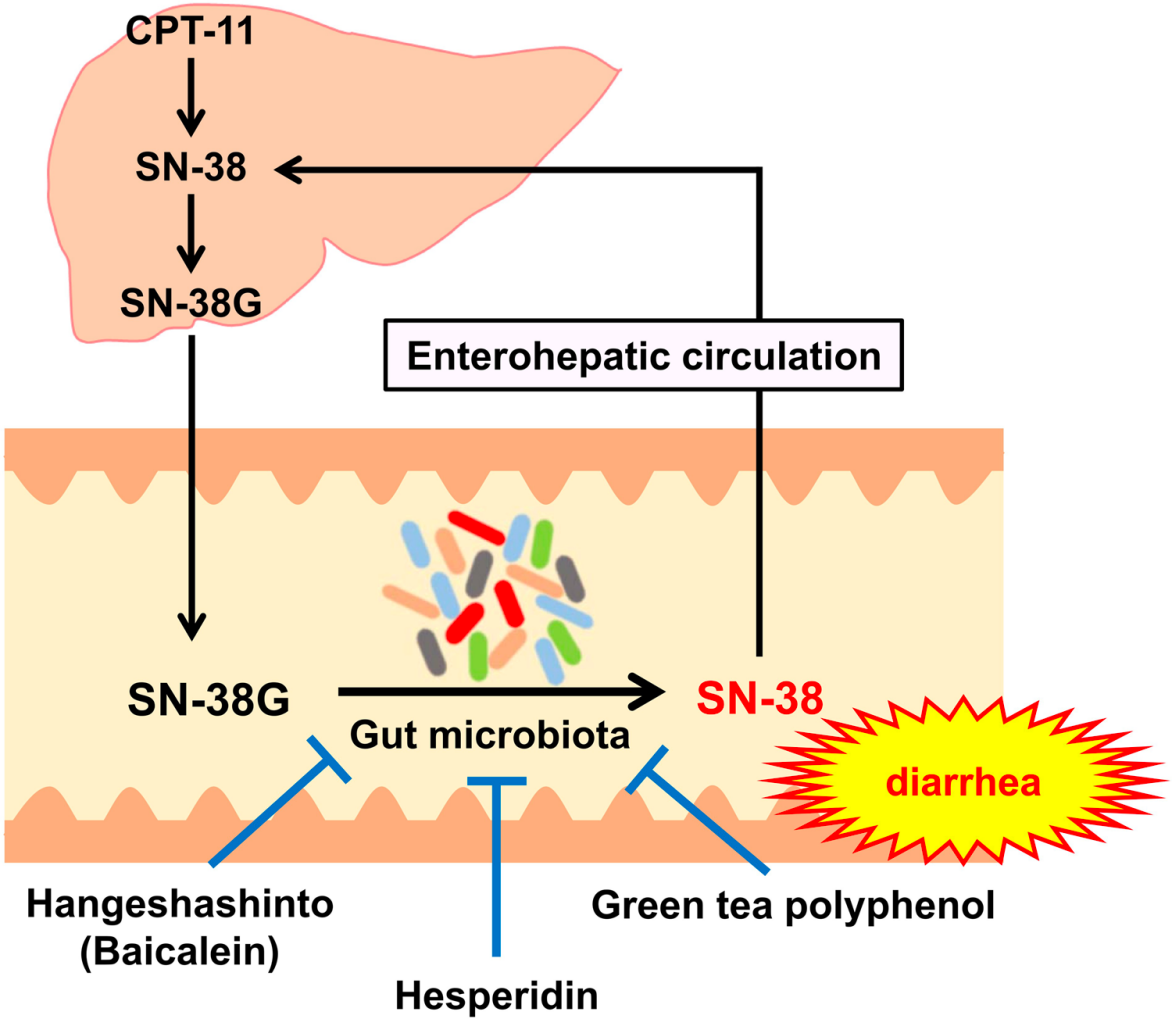
Effect of natural products on CPT-11-induced diarrhea via gut microbiota

Moreover, other natural products have been shown to prevent CPT-11-induced diarrhea via microbiota-mediated mechanisms. Hesperidin, a citrus flavonoid, reduces diarrhea severity and restores the microbial balance, particularly that of *Alistipes*, *Limosilactobacillus*, *Rikenella*, and *Mucispirillum*, in CPT-11-treated models [38]. Green tea polyphenols similarly ameliorate CPT-11-induced diarrhea through the modulation of gut microbial communities in animal experiments [39], highlighting the therapeutic potential of microbiota-targeted natural agents (Fig. 1).

## Additional basic and clinical research on other Kampo formulations

Inchinkoto (ICKT, Yin-Chen-Hao-Tang in Chinese), a three-herb formula composed of *Artemisiae capillaris flos* (Artemisia capillaris flower), *Gardeniae fructus* (Gardenia fruit), and *Rhei rhizoma* (Rhubarb), is prescribed for the alleviation of jaundice. Its choleretic effect arises from enhanced biliary excretion via the upregulation of hepatic multidrug resistance-associated protein-2 (MRP2) transporters, a mechanism ultimately dependent on gut microbial metabolism. Specifically, geniposide from Gardenia fruit is converted into its aglycone genipin by the gut microbiota, which upregulates MRP2 expression and promotes bile flow [40, 41]. The requirement for microbial biotransformation is underscored by the loss of hepatoprotective activity in antibiotic-treated mice [42]. In a clinical investigation, Yamashita et al. examined obstructive jaundice patients and reported that increases in bile flow on days 2 and 3 postadministration were positively correlated with fecal genipin-producing activity. Moreover, genipin production correlated with the abundance of the order Clostridiales (obligate anaerobes) [43], suggesting that the modulation of specific microbial taxa could enhance therapeutic efficacy.

Yokukansan (YKS, Yi-Gan-San in Chinese), indicated for behavioral and psychological symptoms in dementia, contains seven crude drugs: *Glycyrrhizae radix* (Glycyrrhiza), *Bupleuri radix* (Bupleurum root), *Cnidii rhizoma* (Cnidium rhizome), *Atractylodis lanceae rhizoma* (Atractylodes lancea rhizome), *Uncariae uncis cum ramulus* (Uncaria hook), *Angelicae acutilobae radix* (Japanese Angelica root), and *Poria* (Poria sclerotium). Ishida et al. demonstrated that antibiotic treatment in mice markedly reduced plasma glycyrrhetinic acid (GA) levels following YKS administration, indicating the suppression of the gut microbial conversion of glycyrrhizic acid (GL) to GA [44]. The active aglycone GA has anti‐inflammatory and hepatoprotective properties; GL is a Glycyrrhiza‐derived glycoside. Given the widespread inclusion of Glycyrrhiza in Kampo formulas and the established risk of pseudoaldosteronism [45], these findings emphasize that both the efficacy and safety of Kampo medicines may hinge on the composition and functionality of the gut microbiota.

Goreisan (GRS, Wu-Ling-San in Chinese) is a five-herb formula-*Alismatis rhizoma* (Alisma rhizome), *Polyporus* (Polyporus sclerotium), *Poria* (Poria sclerotium), *Atractylodis rhizoma* (Atractylodes rhizome), and *Cinnamomi cortex* (Cinnamon bark)-that improves body water imbalance [46]. Goreisan is used for headache and cerebral edema, and inhibition of aquaporin-4 (AQP4) function is implicated in these effects [47]. Mouse studies suggest that its antidiarrheal action is mediated via changes in bile-acid composition resulting from alterations in the gut microbiota [48]. Metabolomic analysis of urine from Goreisan-treated rats has been performed [49], and analyses targeting the gut microbiota are advancing for various Kampo formulas.

## Future research directions: from basic to clinical studies

The composition and function of the gut microbiota are known to be influenced by drugs, such as antibiotics [50, 51], dietary habits [52–54], and aging [55]. Individual responses to Kampo medicines exhibit considerable variability, and differences in gut microbial profiles likely contribute to this heterogeneity. Through mechanistic studies, elucidating how specific fluctuations in the microbiota affect the pharmacodynamics of Kampo formulations could enable the personalized optimization of these treatments.

Although basic and animal studies have demonstrated the pivotal role of intestinal bacteria in mediating the effects of Kampo formulas, crude drugs, and natural products, clinical reports that directly integrate microbiome analyses with Kampo interventions remain scarce. Limitations in sample size and diversity in study designs constrain the strength of the current conclusions. Future efforts should therefore focus on large-scale, multicenter randomized controlled trials complemented by multiomics approaches that combine shotgun metagenomics and metabolomics to comprehensively interrogate microbiota‒Kampo interactions and translate these insights into precision therapeutic strategies.

## Conclusion

This review comprehensively addressed the mechanisms by which traditional Kampo medicines exert their effects via the gut microbiota, spanning evidence from foundational animal studies to current clinical applications. Recent advances in analytical technologies have propelled gut microbiota research forward, revealing its involvement not only in gastrointestinal disorders but also in neurological conditions and skin physiology. The notion that the pharmacological effects of Kampo formulations, which have been traditionally used, are manifested through interactions with intestinal bacteria is both scientifically intriguing and clinically significant. Continued investigations into the dynamic interplay between Kampo medicines and the gut microbiota hold great promise for advancing both mechanistic understanding and personalized therapeutic applications in the future.
